# A Case of Metastatic Seminoma Mimicking a Primary Pancreatic Tumor

**DOI:** 10.7759/cureus.70329

**Published:** 2024-09-27

**Authors:** Ahmad Alomari, Ismail Althunibat, Mark S Obri, John Curran, Basel Aldroubi, William Davis, Robert Pompa

**Affiliations:** 1 Internal Medicine, Henry Ford Health System, Detroit, USA; 2 Internal Medicine, Saint Michael's Medical Center, Newark, USA; 3 Gastroenterology and Hepatology, Henry Ford Health System, Detroit, USA; 4 Medical College, Tishreen University, Lattakia, SYR

**Keywords:** germ cell tumor, metastatic pancreatic mass, metastatic seminoma, obstructive jaundice, pancreatic lesions

## Abstract

Metastatic seminoma to the pancreas is exceedingly rare, with few reported cases in medical literature. We present a case of a 66-year-old male, six years post-remission from testicular seminoma, who presented with obstructive jaundice and a pancreatic mass mimicking primary malignancy. Diagnostic workup including endoscopic ultrasound-guided biopsy confirmed metastatic seminoma. He underwent successful treatment with four cycles of cisplatin and etoposide, achieving complete remission. This case underscores the diagnostic challenge of pancreatic metastases and emphasizes the role of biopsy in guiding appropriate management. Awareness of such presentations is crucial for timely intervention and improved patient outcomes.

## Introduction

Pancreatic cancer is one of the most aggressive forms of cancer, often diagnosed at an advanced stage with high mortality rates even with treatment [1]. Metastatic cancer of the pancreas is seen in 2-5% of pancreatic malignancies and usually comes from renal cell carcinoma, lung cancer, breast cancer, melanoma, and, in rare instances, seminoma [2,3]. Seminoma is a germ cell tumor of the testicles that rarely metastasizes to the GI tract with an incidence of less than 1% with very few cases involving the pancreas and even rarer, without extensive metastasis [3-7]. We present a rare case of a metastatic seminoma after six years of complete remission mimicking a primary pancreatic tumor and highlight the importance of biopsy and histopathological evaluation. 

## Case presentation

**Figure 1:**
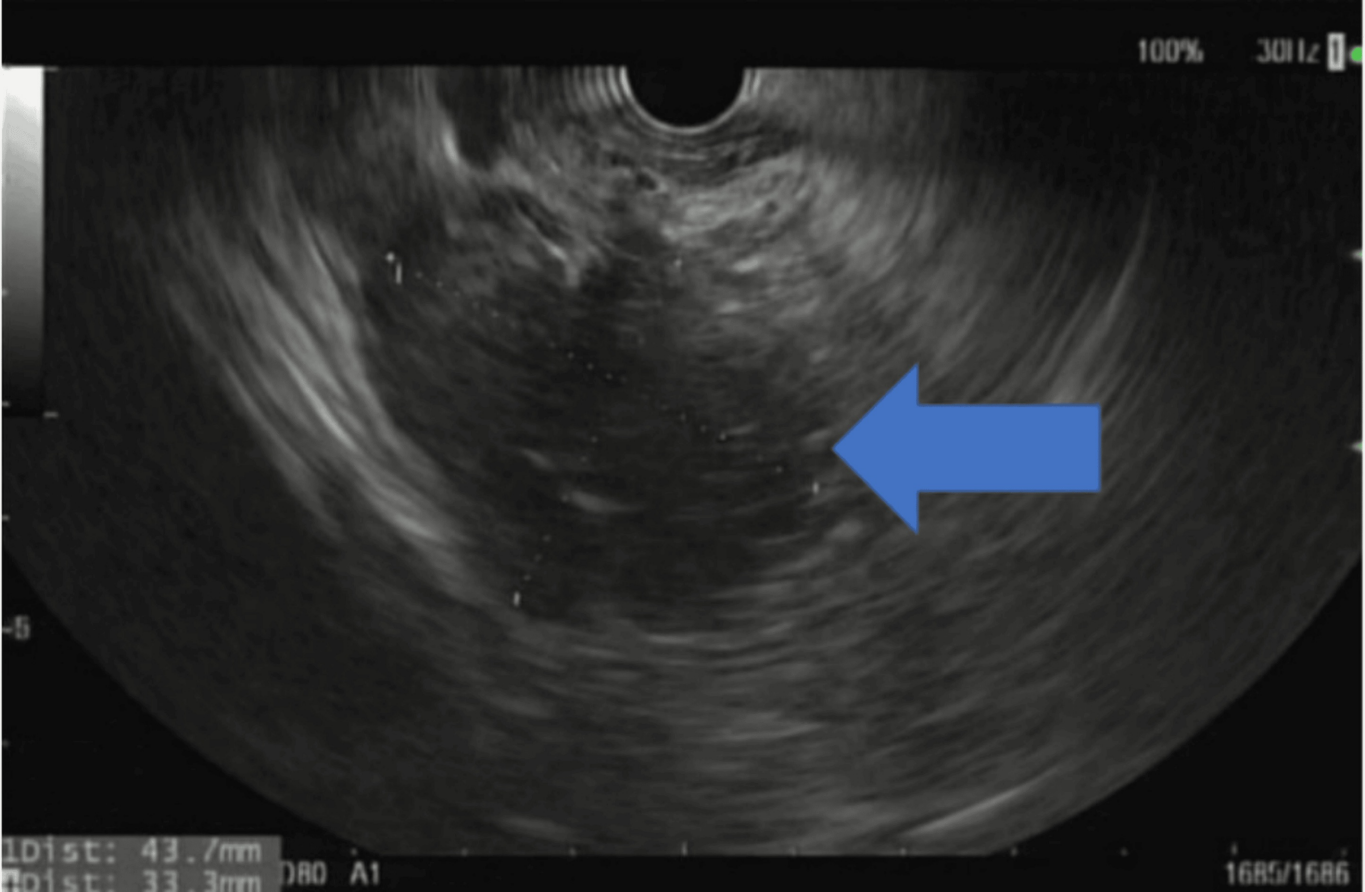
Endoscopic ultrasound showing an oval, hypoechoic, homogenous and solid shaped mass in the pancreatic head measuring 33 mm x 43 mm in maximal cross-sectional diameter (blue arrow).

**Figure 2:**
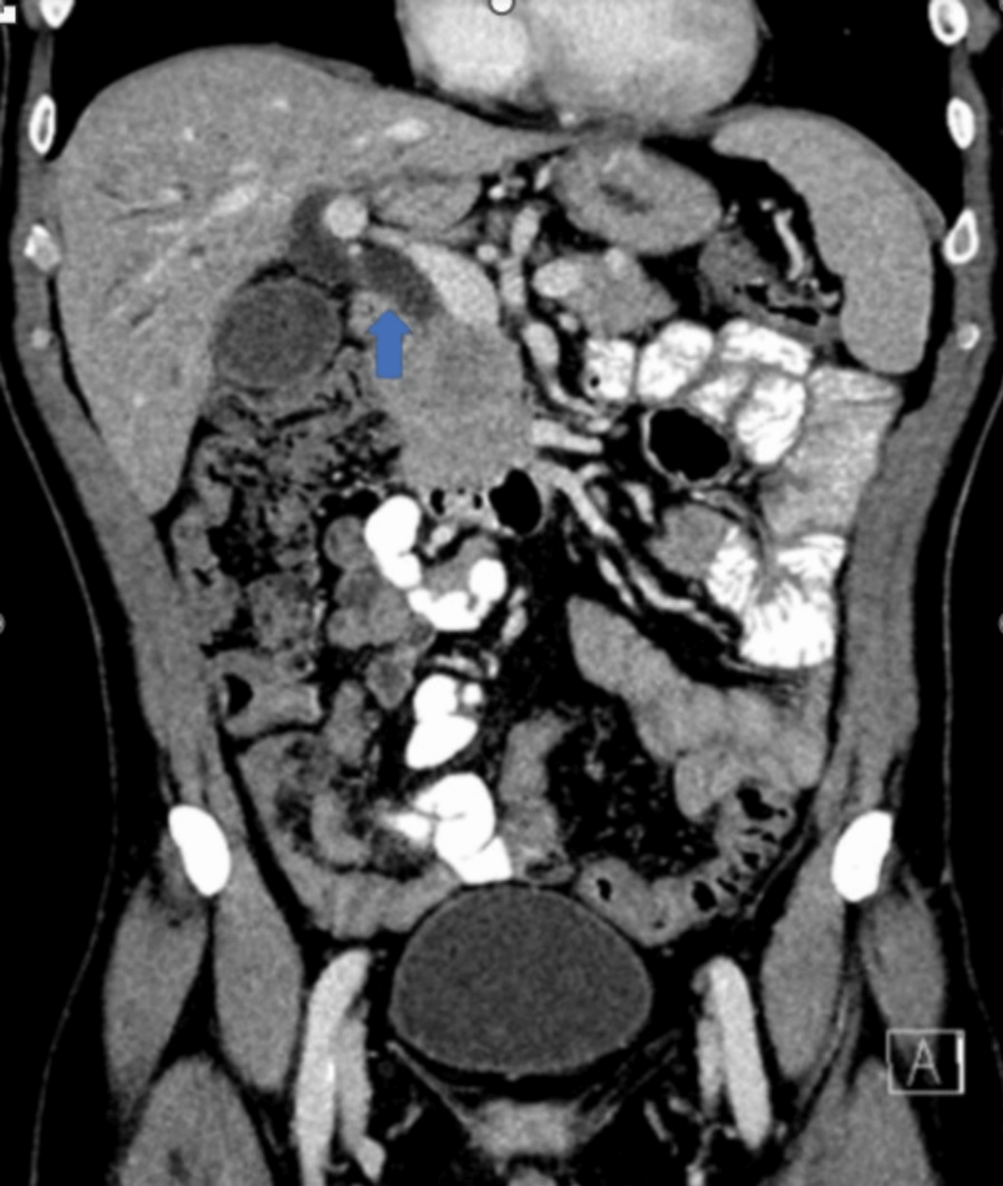
Coronal contrast-enhanced CT image demonstrating marked common bile ductal dilatation of 13 mm (blue arrow). Significant intrahepatic biliary ductal dilatation (not seen on this image) was also noted.

**Figure 3:**
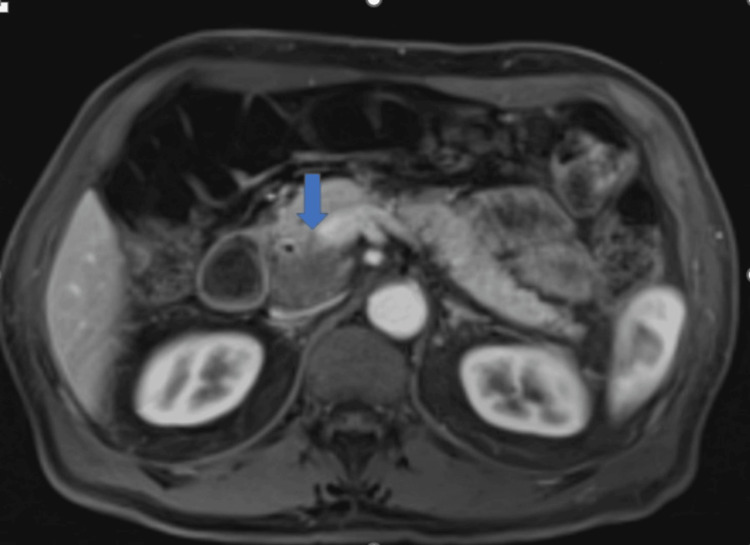
Coronal T2-weighted MRI showing a hypo-enhancing mass in the pancreatic head measuring 51 mm exhibiting restricted diffusion (blue arrow).

A 66-year-old male with a past medical history of testicular seminoma status post bilateral orchiectomy with retroperitoneal lymph node resection, and complete remission for six years without prior chemo or radiotherapy, presented with a chief complaint of itchiness for three weeks prior to presentation, associated with dark-colored urine, subjective yellowing of the skin and mild abdominal pain. His examination was only remarkable for scleral icterus. Labs showed AST 304 U/L, ALT 526 U/L, ALP 287 U/L, and total bilirubin of 8.3 mg/dL with direct bilirubin of 5.3 mg/dL. Ultrasound demonstrated intrahepatic and extrahepatic biliary duct dilatation, with no definitive obstructing lesion visualized (Figure 1). CT scan and MRI showed a mass effect causing biliary dilation with abnormal findings (Figures 2-3). 

The patient denied any history of pancreatic or liver cancer in the past or in his family history and was only notable for a previous history of tobacco use and testicular seminoma. The patient was referred to advanced gastroenterology who performed an endoscopic ultrasound and endoscopic retrograde cholangiopancreatography with biopsy (Figure 2). A mass was identified in the pancreatic head and fine needle aspiration was performed with a single plastic 10 Fr by 9cm stent placed in the common bile duct with subsequent bile flow. 

Subsequent pathology showed neoplastic cells positive for D2-40, OCT3/4, CD117, and negative for CD45, CK7, SOX10, AE1/AE3, and CD30, consistent with his previous primary diagnosis of seminoma. The patient was referred to oncology and subsequently completed a workup with a CT thorax and PET scan showing that the seminoma was only in the pancreas. He completed 4/4 cycles of etoposide and cisplatin as a case of stage III seminoma with a pancreatic mass, achieving remission as no significant uptake within the previously seen pancreatic head was seen on the follow-up PET scan six months later. His obstructive jaundice symptoms have completely resolved and the stent was removed as well.

## Discussion

Testicular seminomas, a subtype of germ cell tumors, tend to metastasize to lymph nodes, liver, lungs, and bones, but pancreatic involvement is rare. A study that involved 94 patients who underwent surgical resection of metastatic lesions of the pancreas showed that only one case was metastatic seminoma while renal cell carcinoma with 21 cases was the most common type found [3].

The clinical presentation of pancreatic metastases from seminoma can mimic primary pancreatic malignancies, as evidenced by the case of a 57-year-old male who presented with obstructive jaundice, abdominal pain, and weight loss with CT revealing a tumor in the head of the pancreas with multiple pathologically enlarged peripancreatic lymph nodes after which a laparoscopic biopsy of the pancreatic lesion and surrounding lymph nodes confirmed the diagnosis of seminoma metastasis [7]. Another case reported a 35-year-old man presented with abdominal pain, pruritus, and 30-pound weight loss. The CT scan showed a heterogeneous pancreatic head mass, biliary dilation, adrenal nodules, and lymphadenopathy. The patient underwent an EUS-guided biopsy of the pancreatic mass and left adrenal with cytology confirming the diagnosis of seminoma [4]. A third case reported a 35-year-old patient diagnosed with metastatic seminoma to the pancreas and vertebra after presenting with back pain and undergoing EUS-guided fine needle aspiration of a pancreatic mass seen on a CT scan [6].

Histopathological examination and immunohistochemical (IHC) staining are important for the diagnosis. Typical markers for seminoma include positive placental alkaline phosphatase (PLAP), OCT3/4, and c-KIT (CD117) [8,9]. Management of metastatic seminoma typically involves systemic chemotherapy, given the high chemosensitivity of germ-cell tumors. The standard regimen includes cisplatin, etoposide, and bleomycin (PEB), which has shown efficacy in achieving a complete response in metastatic seminoma cases [10]. The case discussed demonstrated a complete response to four cycles of PEB chemotherapy, with ongoing remission. This highlights the need for a broad differential diagnosis and the importance of having trained gastroenterologists available for a biopsy of the pancreatic mass. 

## Conclusions

In conclusion, while metastatic seminoma to the pancreas is rare, it should be considered in patients with a history of testicular cancer presenting with pancreatic masses. Accurate histopathological diagnosis and appropriate systemic chemotherapy are critical for good prognosis. 

## References

[1] Ferrone CR, Brennan MF, Gonen M (2008). Pancreatic adenocarcinoma: the actual 5-year survivors. J Gastrointest Surg.

[2] Olson MT, Wakely PE Jr, Ali SZ (2013). Metastases to the pancreas diagnosed by fine-needle aspiration. Acta Cytol.

[3] Reddy S, Edil BH, Cameron JL (2008). Pancreatic resection of isolated metastases from nonpancreatic primary cancers. Ann Surg Oncol.

[4] Mai D, Krinsky M, Jagannath A (2020). Unusually yellow for a young fellow: a case of metastatic seminoma mimicking pancreatic adenocarcinoma. Am J Gastroenterol.

[5] Dong W, Gang W, Liu M, Zhang H (2016). Analysis of the prognosis of patients with testicular seminoma. Oncol Lett.

[6] Stoos-Veic T, Tadic M (2014). Endoscopic ultrasound and fine needle aspiration for the diagnosis of extragonadal seminoma metastatic to the pancreas. Endoscopy.

[7] Wehrschütz M, Stöger H, Ploner F (2002). Seminoma metastases mimicking primary pancreatic cancer. Onkologie.

[8] Senadhi V, Dutta S (2012). Testicular seminoma metastasis to the gastrointestinal tract and the necessity of surgery. J Gastrointest Cancer.

[9] Tourne M, Radulescu C, Allory Y (2019). Testicular germ cell tumors: Histopathological and molecular features [article in French]. Bull Cancer.

[10] Lamichhane A, Mukkamalla SKR (2023). Seminoma. StatPearls.

